# Mr. Wynne's Case of Cured Hydrophobia

**Published:** 1813-04

**Authors:** Rice Wynne

**Affiliations:** Shrewsbury


*To the Editors of the Medical and Physical Journal.*

GENTLEMEN,

THERE are not, I believe, any means established by which we are enabled even to mitigate the horrid and truly pitiable sufferings consequent to the bite of a rabid animal. Happily, however, two cases of Hydrophobia successfully treated, by Mr. Tymon and Dr. Shoolbred, have lately been transmitted to us from the East Indies. I likewise feel the utmost satisfaction in relating the successful issue of the case of a man in my own neighbourhood, who was bitten by a rabid dog, and which bears the strongest resemblance to the recorded symptoms of Hydrophobia. I do not here mean to enter into a detailed account, as I intend soon to publish every particular connected with the appearance of the dog, the injury he committed, as well as the subsequent illness, and treatment, of the man who was bitten. I therefore hope the following statement will for the present be deemed satisfactory.

Abraham Cooke, who resides at Atcham, about four miles distant from Shrewsbury, was, on the 22d of January last, bitten in the hand by a pointer bitch, bearing evident signs of being mad. By the persuasion of his friends, he almost immediately set out and walked to Shrewsbury. On his arrival, the bitten part was excised by Mr. Thomas Sutton, surgeon. The wound soon healed, and he continued in good health and spirits, always making light of the accident, until Friday the 5th of February, when, in the morning, he first began to feel and complain of an uneasiness and soreness in that part of his hand where he had received the injury. On Saturday and Sunday it became gradually worse; and on Monday morning, after a restless night, he arose with increased pain and soreness in his hand, attended with headache, sickness, and oppression at the pit of his stomach: his breathing breathing was difficult: notwithstanding, he went to his work, but very soon became much worse. He was prevailed upon to drink some warm beer, and was immediately seized with violent vomiting. He with difficulty reached his home, and on his way was much distressed, as he believed the people who were passing were determined to ride over him. His wife, seeing him so ill, pressed him to take some water; he shewed a great dread of it, and could not be prevailed upon to drink any, assigning, as the reason of his objection, the pain and vomiting occasioned by swallowing the beer. She then procured some surfeit-water, to which he made the same objection; she put it to his mouth, but could not say whether any was swallowed; if any, it must have been a very small quantity; she was terribly frightened at his looks. All the symptoms rapidly increased: his eyes were inflamed and staring; his face was likewise inflamed, and his features were contorted, and indicated the greatest dread and anxiety. It was with difficulty he was detained in his bed, and he appeared to be watching, and anxious to escape some object that occasioned his distress.

At this time (about one o'clock, p. m.) I was passing through the village, and was desired to visit him. I found him in the situation related. In a very short time afterwards, his left hand, arm, and head, were convulsed. I waited to hear the history of the accident from his wife, and then pressed him to drink some water; I could not prevail, and, although I did not observe any additional horror at the moment I offered it, still it was evident he was too much agitated to be able to drink. I was truly anxious for the advice and assistance of my much-esteemed friend, Mr. Thomas Sutton; at the same time I was aware that the delay of an hour might hazard the life of our patient. I therefore had recourse to the abstraction of a large quantity of blood, and allowed it to flow until he fainted. He remained for near an hour with scarcely a perceptible pulse; and it was evident the whole time his disease was abating. His countenance became composed, and much paler; his eyes were less inflamed; the convulsions ceased; and, to my complete satisfaction, he enquired if he might drink some water; and, when it was brought to him, he seemed to enjoy it. I now left him, desiring that, if any return of his disease took place, I might be immediately acquainted with it. I sent him large doses of opium, with calomel, and James's powder, adhering, as closely as possible, to the successful plan adopted by Dr. Shoolbred, and communicated in the Medical and Physical Journal for January, 1813.

On Tuesday morning, at seven o'clock, I again visited him.

He had slept the early part of the night, but had been disturbed with horrid dreams; at the same time he told me, "he had not been half so much alarmed by his dreams, as with the appearance of the dog in his room the day before, and which did not leave the room until he fainted from the bleeding. He seemed agitated, and said he was dreadfully ill, and should never sleep again. There were convulsive startings in his hand, wrist, and shoulders. He told me he thought there was something alive in his wrist; he refused to take either coffee or water. His countenance was composed, but thoughtful; he said his neighbours had been making a noise on purpose all night, and every thing went through his brains. He started at the slightest sound or motion; and so quick was his sense of hearing, that he knew, by the sound, to whom a waggon that was passing by belonged. I considered it necessary to repeat the bleeding, and the result was exactly the same as before described—he fainted; and from that time was perfectly composed, and free from convulsion, until about three o'clock, p. m. when his wife thought for a moment she observed a twitching in his shoulder.

On Wednesday, I saw him in a comfortable state.—On Thursday, the 11th, after sitting up for some time, he was much fatigued, and fainted. I was sent for, and upon my arrival his wife said, "she was sorry I had been troubled to come, as the fit was nothing like what I had seen before, and was not more than such as he had been accustomed to when he had been drinking hard, or was much tired, and that it was against her consent that the messenger came." I was satisfied, from appearances, she was right; and I left him without any apprehension. Since that time I have regularly visited him, and there has not been one untoward symptom. He is now apparently well, excepting that his mouth is sore from the effect of the mercury.

I here wish to mention, that, immediately on my return from Atcham on Monday, the 8th, the first afternoon I saw the patient, I called upon Mr. Thomas Sutton, and requested him to take the earliest opportunity of observing the case, and begged that he would have the kindness to suggest any thing that he thought likely to be of service. He and Mr. Sutton, as well as others of the profession, have been present.

I remain, Gentlemen,

*Shrewsbury,* Your obedient Servant,

